# PhLeGrA: Graph Analytics in Pharmacology over the Web of Life Sciences Linked Open Data

**DOI:** 10.1145/3038912.3052692

**Published:** 2017-04

**Authors:** Maulik R. Kamdar, Mark A. Musen

**Affiliations:** Center for Biomedical Informatics Research, Stanford University, USA; Center for Biomedical Informatics Research, Stanford University, USA

**Keywords:** graph analysis, federated querying, data mining, semantic web, drug–drug interactions

## Abstract

Integrated approaches for pharmacology are required for the mechanism-based predictions of adverse drug reactions that manifest due to concomitant intake of multiple drugs. These approaches require the integration and analysis of biomedical data and knowledge from multiple, heterogeneous sources with varying schemas, entity notations, and formats. To tackle these integrative challenges, the Semantic Web community has published and linked several datasets in the Life Sciences Linked Open Data (LSLOD) cloud using established W3C standards. We present the PhLeGrA platform for Linked Graph Analytics in Pharmacology in this paper. Through query federation, we integrate four sources from the LSLOD cloud and extract a drug–reaction network, composed of distinct entities. We represent this graph as a hidden conditional random field (HCRF), a discriminative latent variable model that is used for structured output predictions. We calculate the underlying probability distributions in the drug–reaction HCRF using the datasets from the U.S. Food and Drug Administration’s Adverse Event Reporting System. We predict the occurrence of 146 adverse reactions due to multiple drug intake with an AUROC statistic greater than 0.75. The PhLeGrA platform can be extended to incorporate other sources published using Semantic Web technologies, as well as to discover other types of pharmacological associations.

## 1. INTRODUCTION

The “Semantic Web” vision of the World Wide Web Consortium (W3C) has provided a unique opportunity towards web-scale computation, seamless integration of big data and structured querying of multiple heterogeneous sources simultaneously. Semantic Web technologies can be used to develop refined approaches to address complex, biomedical challenges, where traditional computational methods are not scalable. However, the structural heterogeneity of the Semantic Web makes the task of serendipitously discovering implicit associations illusive. In this paper, we present the PhLeGrA platform – Linked Graph Analytics in Pharmacology. The PhLeGrA platform provides an approach to tackle the structural heterogeneity in the biomedical Semantic Web and discover newer pharmacological associations.

### 1.1 Systems Pharmacology

Adverse drug reactions (ADR) often result in the hospitalization or serious injury of more than 2 million individuals in the United States, with more than 100,000 deaths annually [27]. Hence, ADRs are the 4^*th*^ leading cause of death ahead of diabetes, AIDS, and pneumonia [8]. The costs of drug-related morbidity and mortality in the United States alone was estimated to be US$177.4 billion in 2000, and has been rising ever since [13]. A majority of these ADRs are caused due to polypharmacy, a situation where multiple concomitant drugs are administered to one patient in a short span of time to treat multiple medical conditions [9]. Drug–drug interactions (DDI) due to polypharmacy are potentially avoidable, if detected early [36].

Post-marketing surveillance is carried out to detect unanticipated DDIs and ADRs. Several studies, which often use the US Food and Drug Administration (FDA) Adverse Event Reporting System (FAERS) [16] or electronic medical records [19], have inferred new DDIs and the ADRs that manifest on the account of those interactions. However, these studies do not systematically demonstrate how the drugs interact within the biological system of the patient, leading to a particular adverse reaction. “Mechanism-based prediction” of DDIs and ADRs can provide a better understanding of the underlying biological mechanisms behind the DDIs [2]. Moreover, this understanding can lead clinicians to prescribe drugs that can cure the same medical conditions in a patient while minimizing the risk of DDIs due to different mechanisms of those drugs.

Newer approaches of integrative pharmacology, termed “systems pharmacology”, are required to attain the objective of mechanism-based prediction and evaluation of DDIs and ADRs [2]. These approaches rely on an exhaustive *systems network*. Such a network must possess knowledge on the drug-induced perturbations of the physiological functions in a biological system as well as knowledge on the underlying biological interactions (e.g. metabolic pathways). However, the data and knowledge to generate such a network exists in several databases and knowledge bases that may be fragmented across the Web. These sources, if available for download, may: *i)* use varying schemas to structure the data, *ii)* use different entity notations (e.g. Proteins referenced using HGNC [31] or KEGG [25] identifiers), and *iii)* use different formats (e.g. XML, CSV, etc.). An *ad hoc* integration approach by downloading and integrating each source independently, and performing manual entity reconciliation and disambiguation, is non-trivial, non-scalable and is often redundant for different tasks.

### 1.2 Semantic Web Technologies

The Semantic Web was conceived with the vision that a decentralized, distributed and heterogeneous data space, extending over the traditional Web, can reveal hidden associations that were not directly observable [5]. Any domain user can query this Web of Data, often called Linked Open Data cloud [6], without being concerned about the underlying heterogeneity and representation. Due to the challenges of integrative bioinformatics, biomedical researchers have been the earliest adopters of Semantic Web technologies and linked data principles to create the Life Sciences Linked Open Data (LSLOD) cloud [7]. Semantic Web technologies include the W3C standards Resource Description Framework (RDF) [26] and the SPARQL graph query language [32]. Biomedical data and knowledge sources are converted to graphs using the triple-based RDF model. SPARQL can use specific expression patterns, termed **triple pattern fragments** (TPF), to query these RDF graphs.

Substantial work has been carried out to publish and link biomedical data and knowledge in the LSLOD cloud by several different efforts [10, 20]. Several sources that may be relevant to systems pharmacology, such as the Comparative Toxicogenomics Database [12] and DrugBank [38], are made available through the LSLOD cloud. However, the task of serendipitously discovering hidden associations from the LSLOD cloud is still non-trivial, and far from complete. We define the term **association** as a mapping between a set of *inputs* and an *outcome*. Hence, the indication that multiple drugs may interact to cause an adverse drug reaction is an association ({*Drug*}_*n*_ → *ADR*).

**Figure 1:**
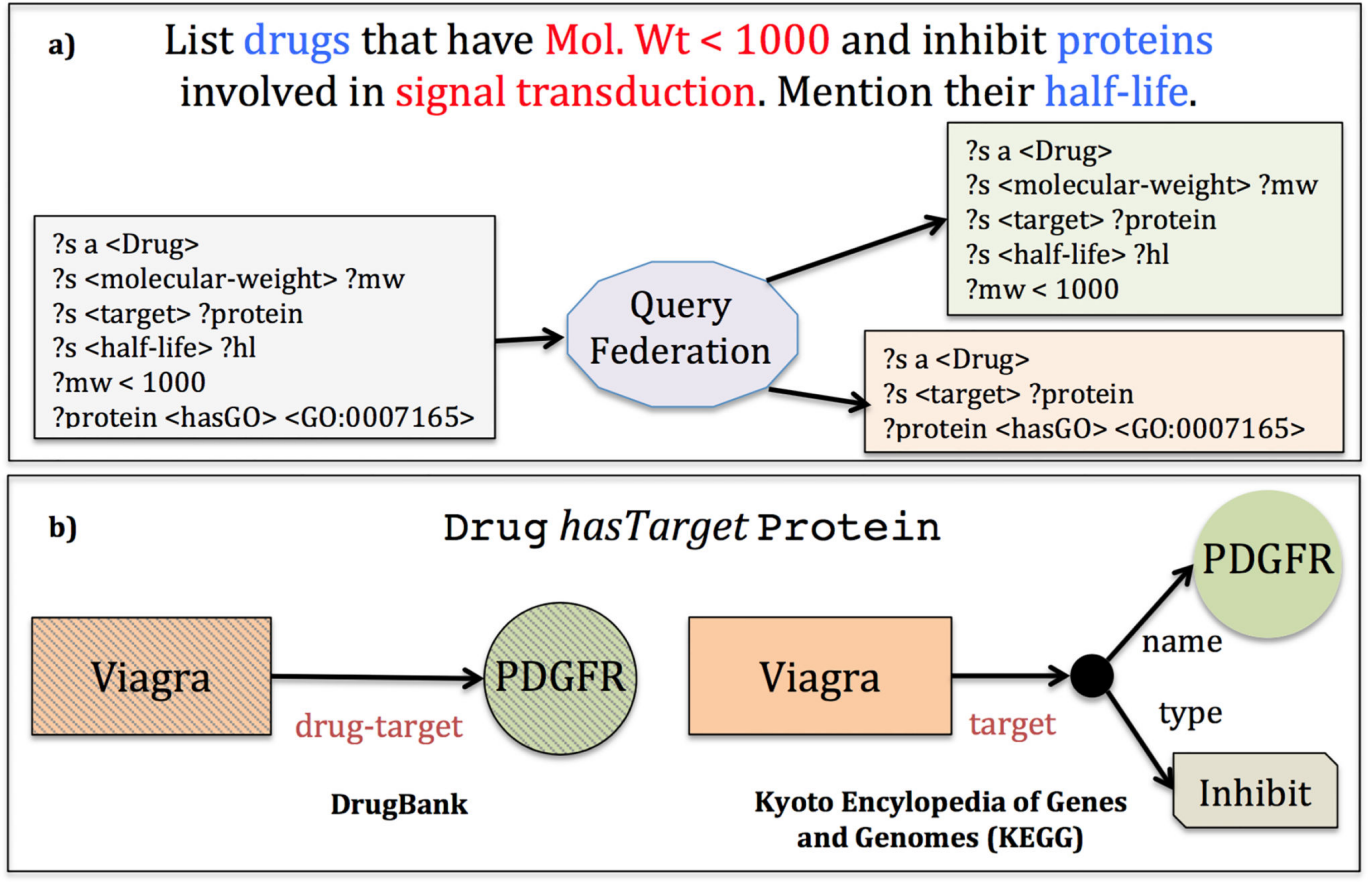
SPARQL Query Federation: a) Methods: Each Triple Pattern Fragment in the SPARQL query is evaluated for each source, before federation. b) Challenges: Different RDF graphs may use different semantics (e.g. *drug-target* and *target*). Different graph patterns may be used to depict the same relation, while capturing additional details.

Biomedical RDF graphs still exist either as RDF data dumps, or are exposed through isolated SPARQL endpoints on the web. Querying multiple isolated SPARQL endpoints simultaneously over the web requires a scalable SPARQL **query federation** method [35]. An example of the process of a query federation method is shown in Figure 1a. Generally, the method evaluates each TPF in a SPARQL query precisely and queries the relevant source where the TPF may exist, before reconciliation of entities and relations.

In some cases, the same relation may be expressed in different RDF graphs using different semantics, or using different graph patterns entirely (e.g. Figure 1b). A user who wishes to aggregate such relations from multiple graphs must be aware of the underlying semantics and the data model. One of the key principles for Linked Data is the use of HTTP-derefenceable Uniform Resource Identifiers (URIs) for **entity reconciliation**. Empirically, we observe that most data publishers create their own URIs to represent entities. In the pharmacological domain, the same drug may be represented using different URIs in different sources and they need to be reconciled during retrieval. This is not possible through current query federation methods.

Using best principles to simply link all data and deploying a robust querying infrastructure is however not sufficient for association discovery. An **analytics** framework that uses the linked data to compute the probability of an association between two types of entities (*inputs* → *outcome*) in different data sources is required. The framework needs to deal with the facts that, *i)* there may be intermediate entities on a path for which there are no observed data, and *ii)* a combination of *inputs* may be associated with an *outcome*.

In this paper, we present the **PhLeGrA**^1^ platform – Linked Graph Analyics in Pharmacology. The PhLeGrA platform combines graph analytics with query federation over the LSLOD cloud to discover hidden associations between entities that have no explicit relations. The key contributions of this research can be described as follows:

1. We develop a pattern-based query federation method over the Web of Linked Data, and demonstrate the extraction of a *k*-partite network composed of distinct entities and relations from multiple sources.
2. We propose and implement a graph analytics method, based on Hidden Conditional Random Fields (HCRF) [33], to discover implicit associations between the different entities in the *k*-partite network.
3. We develop a provenance-enabled visualization interface that allows a user to search and explore the interconnecting paths between drugs and ADRs.
4. Finally, we critique on the current state of the LSLOD cloud and discuss the challenges encountered while mining the LSLOD cloud to discover new associations.

The paper is organized as follows: **Section 2** gives a brief overview on biomedical projects that use Semantic Web technologies. **Section 3** outlines the methodology used for query federation and graph analytics framework. **Section 4** lists the set of data sources used for developing a prototype of the PhLeGrA platform. **Section 5** presents the results of our prototype. Finally, in **Section 6** we discuss the limitations of our approach and challenges faced.

All results and methods of this paper, as well as all developed visualization tools, are available online at: http://onto-apps.stanford.edu/phlegra.

## 2. RELATED WORK

Entity reconciliation in the biomedical domain is a major problem, as there is often no agreement on a unique representation of a given entity. Many biomedical entities are referred to by multiple labels, and same labels may be used to refer different entities. To resolve this problem, efforts such as Bio2RDF [10] and Linking Open Drug Data [20] have released guidelines for using *x-ref* attributes rather than using the same URI. Similar entities in different sources are mapped to each other, or all similar entities are mapped to a common terminology using *x-ref* attributes [29].

Most query federation methods do not take *x-ref* attributes into account, and rely only on the URIs of the entities. Federation engines may use an index linking all possible URIs to a particular string term, but such an index can be difficult to maintain [35]. Rule-based federation engines, on the other hand, can use a set of ‘patterns’ to determine which SPARQL endpoints and URIs to query for a particular class (e.g. Drug) or an entity (e.g. Lepirudin) [22]. However, only semi-automated methods can generate such patterns to query for a particular class in different sources. Generating patterns to query similar entities requires significant manual intervention [17], and is tedious.

There has been a lot of research to predict DDIs, or predict ADRs that manifest due to concomitant intake of multiple drugs, by mining spontaneous reporting systems such as FAERS or electronic medical records [16, 19]. Systems pharmacology methods have also been explored in the context of drug-ADR association discovery or drug repurposing (use of existing drugs to treat new conditions) [2]. These methods generally combine databases and knowledge bases manually without the use of Semantic Web technologies. *CauseNet* combines four biomedical sources into a *k*-partite network for generating new drug repurposing hypotheses [28]. While this approach is similar to our approach, we argue that our query federation method over the LSLOD cloud will be faster, and help generate such *k*-partite networks easily.

Recently, there has been some research to leverage the LSLOD cloud for discovering new DDIs. *Tiresias* processes various sources of drug-related data and knowledge as inputs and predicts new DDIs using large-scale similarity matching [14]. The Translational Ontology-anchored Knowledge discovery Engine (*TOKEn*) evaluates induced associations between proteins and phenotypes, using ontological hierarchies and DrugBank [38], to find drugs for skin cancer [34]. However, most approaches consider binary drug pairs and not multiple drug interactions [4], they ignore the underlying molecular mechanisms, and they may not associate the adverse drug reactions with the DDIs [3].

## 3. METHODS

**Figure 2:**
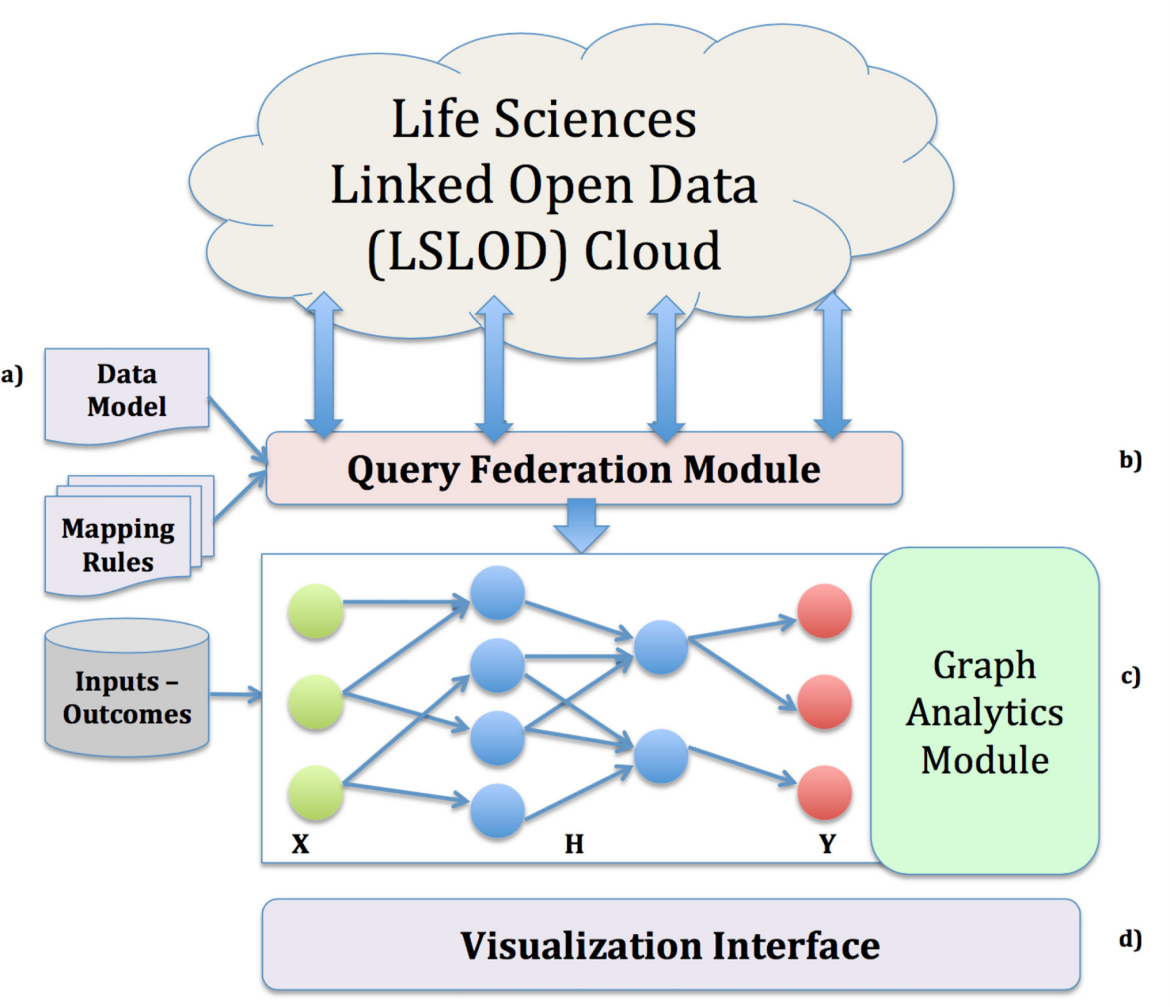
Platform for Linked Graph Analyics in Pharmacology (PhLeGrA). Using the Data Model (a) and mappings rules, the query federation module (b) extracts a *k*-partite HCRF network from the LSLOD Cloud. It uses an external database of *inputs* and *outcomes* to predict the probabilities of associations (c). A visualization interface allows the domain user to navigate the *k*-partite network (d).

The PhLeGrA platform relies on a data model that captures all the relevant pharmacological relations required for developing a systems pharmacology network (**Section 3.1**). This data model is used by our query federation method (**Section 3.2**) to retrieve entities and relations from multiple sources in the LSLOD cloud and populate our *k*-partite network. Our analytics framework inspired from Hidden Conditional Random Fields performs inference over this *k*-partite network (**Section 3.3**). The query federation method and the graph analytics framework are bundled in the architecture of the PhLeGrA platform (Figure 2).

### 3.1 Data Model

**Figure 3:**
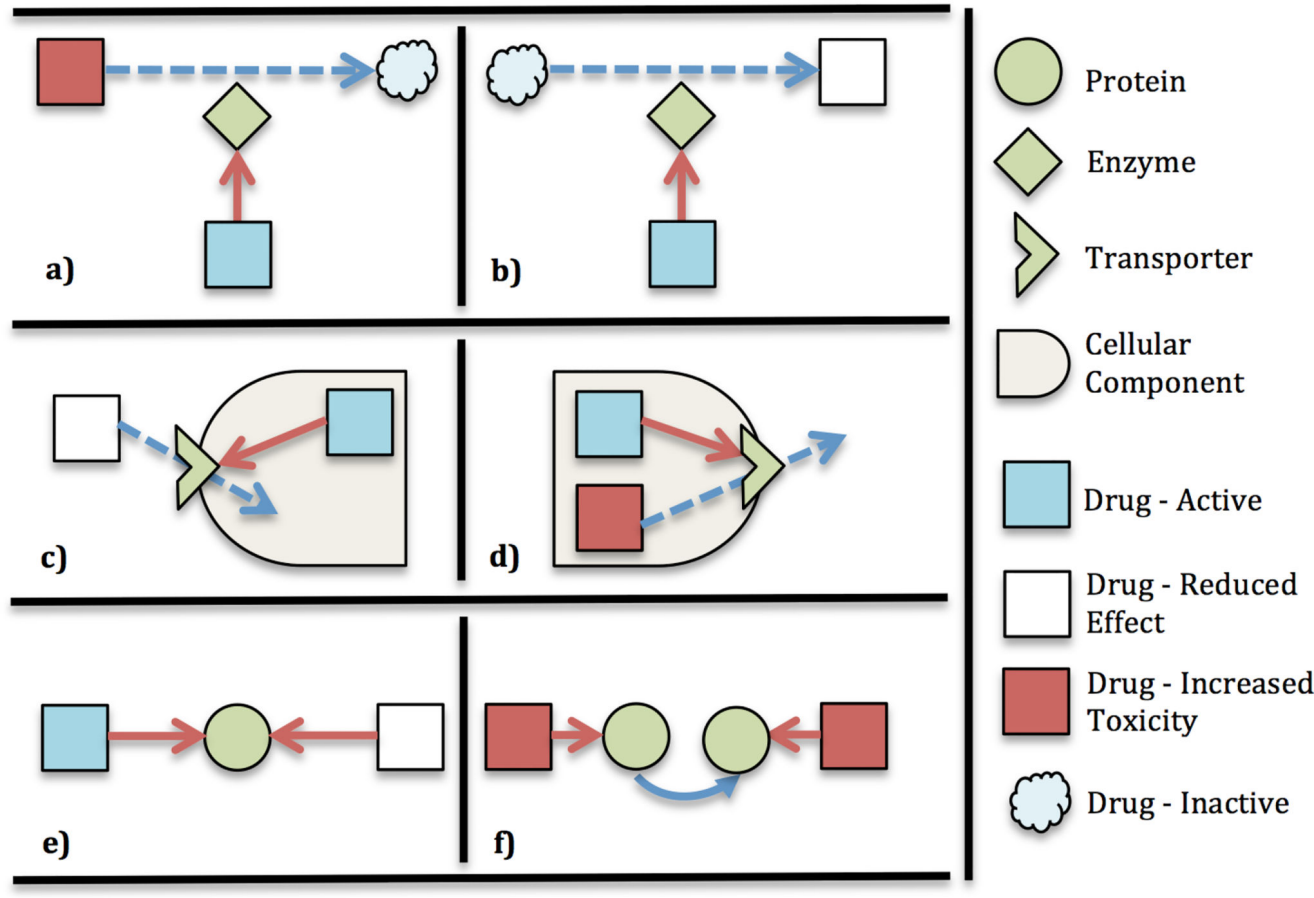
Several underlying mechanisms for drug–drug interactions. a, b) The inhibition of enzymes that metabolize a drug to its inactive or active state. c, d) The inhibition of transporters can decrease the absorption or elimination of a drug. e) Two drugs target the same protein to reduce the effect of one drug. f) Two drugs target proteins in the same pathway to increase the effect of both drugs.

Our data model aims to provide an abstract representation of the molecular mechanisms behind DDIs in the biological system of a patient. There are several underlying mechanisms through which two drugs can interact [21]. For example, most drugs are metabolized to their inactive or active forms by particular proteins^2^, termed enzymes. When the expression of these enzymes is inhibited by another drug, this can lead to increased toxicity (Figure 3a) or decreased effect (Figure 3b) of the former drug respectively. Inhibition of the expression of drug transporters (specialized proteins) can alter the absorption (Figure 3c) and elimination (Figure 3d) of drugs in the body. Different drugs can target the same protein resulting in either an additive or a negative effect (Figure 3e). A pathway is a series of actions among proteins in a cell that leads to changes in the cell or production of other proteins. A drug targeting an upstream protein can affect the activity of another drug targeting a downstream protein in a pathway (Figure 3f).

**Figure 4:**
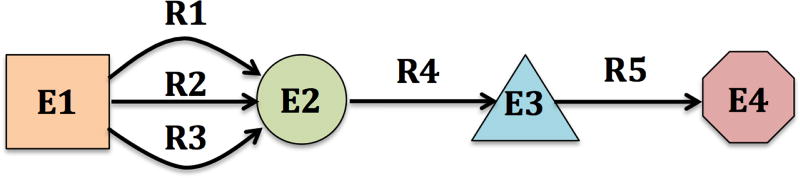
A visual depiction of the data model used for generating a *k*-partite network

We simplify these mechanisms to a more abstract representation. We have four different types of biological entities — **(E1)** Drug, **(E2)** Protein, **(E3)** Pathway, and **(E4)** Phenotype (adverse drug reaction). We also have five different types of biological relations — **(R1)** Drug *hasTarget* Protein, **(R2)** Drug *hasEnzyme* Protein, **(R3)** Drug *hasTransporter* Protein, **(R4)** Protein *isPresentIn* Pathway, and **(R5)** Pathway *isImplicatedIn* Phenotype. The entities and relations, retrieved from the LSLOD cloud, form a *k*-partite network — a network whose nodes can be partitioned into *k* different independent sets (*k* = 4). A visual depiction of the model is shown below, in Figure 4.

### 3.2 Query Federation

During query federation, SPARQL queries are decomposed into Triple Pattern Fragments (TPF) and each fragment is executed individually across several sources (Figure 1a). This decomposition of SPARQL queries can be governed through mapping rules [22]. PhLeGrA uses a modified TPF query engine [37] with the inputs: *i)* the set of SPARQL endpoints, *ii)* the data model, and *iii)* mapping rules.

A mapping rule, in this work, maps an entity type (e.g. **E1**) or a relation type (e.g. **R1**) in our data model to a graph pattern observed in an RDF graph, if the relevant element or relation exists in the graph. For example, DrugBank [38], a data source that contains information on drugs, contains entities of type **E1** ( Drug) and relations of type **R1** ( Drug *hasTarget* Protein). Then, the graph patterns observed in DrugBank RDF graph are mapped as follows:

$$E1≔?E1\overset{\text{rdf}:\text{type}}{\to }\text{drugbank}:\text{Drug}$$

$$R1≔?E1\overset{\text{drug}}{\leftarrow }\text{drugbank}:\text{Target-Relation}\overset{\text{target}}{\to }?E2$$

These mapping rules are manually curated by observing the vocabularies of the LSLOD sources used in our prototype. These mapping rules are described using an extension of the Vocabulary of Interlinked Datasets (VoID) [1, 17]. They are used by our query federation module to populate the *k*-partite graph from the LSLOD sources.

The query federation module also deals with reconciliation of similar entities expressed using different URIs in different RDF graphs. For each entity, the module collects specific *x-ref* attributes provided by the biomedical data publishers. These attributes may link similar entities in different graphs to each other, or may link similar entities to a unique term in a designated terminology. For instance, retrieving information on the drug Lepirudin and the protein Prothrombin from two sources requires different patterns: drugbank:DB00001 → kegg:D06880 and drugbank:BE0000048 → hgnc:3535 kegg:HSA_2147. In the latter case, the different proteins are mapped to the Hugo Gene Nomenclature Committee terms (HGNC) [31].

To generate the *k*-partite network, the federation module queries all sources simultaneously in the following order:

1. Retrieves all the entities of a given type (e.g., **E1**), and generates new nodes in the *k*-partite network.
2. Retrieves relevant *x-ref* attributes for each entity.
3. Reconciles entities that are mapped to the same term in a given terminology (e.g. HGNC), or are mapped to each other using *x-ref* attributes.
4. Retrieves all relations of a given type (e.g. **R1**) among entities of two types (e.g., **E1** and **E2**), and generates edges between the nodes in the *k*-partite network.
5. Detect the largest connected component (treating the network as undirected)

The nodes and edges are also annotated with a list of data sources from which they were retrieved for provenance.

### 3.3 Hidden Conditional Random Field

The primary goal of PhLeGrA is to discover associations between a set of *inputs* and an *outcome*, i.e. the probability an *outcome* (ADR) is observed considering the *inputs* (drugs). The graph analytics module in PhLeGrA (Figure 2c) takes as input the extracted *k*-partite network. As we are predicting a structured *outcomes* vector **y** using a structured *inputs* vector **x**, the *k*-partite network is represented as a conditional random field. A conditional random field is a type of a discriminative undirected probabilistic graphical model, commonly used in machine learning for structured prediction. As we assume the state of the intermediate entities (e.g. Protein) on the path from *inputs* and *outcomes* (the end layers in the *k*-partite network) will be unobserved, our model is actually a (*k* − 2)-layer hidden conditional random field (HCRF). An HCRF framework learns a set of unobserved variables, and makes no assumption on the independence of the *inputs* [33]. Instead of using a simple Bayesian directed probabilistic model, we made a decision choice towards HCRF to make our probabilistic model more scalable, to allow structured *outcome* prediction, and to incorporate the concept of unobserved entities.

The graph analytics module generates joint probability distributions over each edge joining nodes of two different entity types (e.g., **E1** and **E2**) in the *k*-partite network. These probability distributions are learnt using an *inputs*–*outcomes* database (described in **Section 4.2**).

The module takes an *inputs* vector **x** — a vector where **x**_*i*_ = 1 if the *i*^*th*^ drug is prescribed to a patient, and outputs an *outcome* vector, where **y**_*j*_ = 1 if the *j*^*th*^ adverse reaction is observed, or zero otherwise. In the following equations, we summarize the original algorithm for learning the parameters of an HCRF by Quattoni, et al. [33].

$$\Psi (y,h,x;\theta )=\sum_{(j,k)\in E}\sum_{l\in L}f_{l}(j,k,y,h_{j},h_{k},x)\theta_{l}$$

$$p(y|x,\theta )=\sum_{h}P(y,h|x,\theta )=\frac{\sum_{h}e^{\Psi (y,h,x;\theta )}}{\sum_{y\prime ,h}e^{\Psi (y\prime ,h,x;\theta )}}$$

$$L(\theta )=\sum_{i}\text{log }P(y_{i}|x_{i},\theta )-\frac{1}{2\sigma^{2}}{\left\|\theta \right\|}^{2}$$

$$∴L_{i}(\theta )=\text{log }P(y_{i}|x_{i},\theta )=\text{log }(\frac{\sum_{h}e^{\Psi (y,h,x;\theta )}}{\sum_{y\prime ,h}e^{\Psi (y\prime ,h,x;\theta )}})$$

$$∴\theta \leftarrow \theta +\alpha *\nabla_{\theta }L(\theta )$$

*P*(**y**|**x**, *θ*) indicates the probability **y** is observed given a set of *inputs* **x**. These are calculated over all possible states for the observations of the hidden nodes **h**. Our goal is to maximize *L*(*θ*), where *θ* represents the parameters of our model. Ψ is a potential function that relies on the edge features of our *k*-partite graph — *E* represents the set of edges and *L* represent the set of states of the connected nodes (*h*_*j*_ = *a, h_k_* = *b*) and *f_l_* is a feature vector based on the configuration *l*. We introduce a regularization term *σ*^2^ that is the variance of *θ*, to avoid overfitting.

The graph analytics module use stochastic gradient ascent to learn the parameters, by iterating over each entry in an *inputs*–*outcomes* database. The parameters are updated on each iteration by using a step rate *α*. The probabilities are calculated using loopy belief propagation.

## 4. DATA

### 4.1 Linked Open Data Sources

The Life Sciences Linked Open Data Cloud (LSLOD) contains several data sources that are relevant to this problem. We integrate four different data sources that are published by the Bio2RDF project (Version 4) [10]:

**D1: DrugBank** [38]: A bioinformatics data source that has comprehensive drug and drug target information
**D2: PharmGKB** [18]: A manually-curated knowledge-base that summarizes protein–drug–disease relations from a literature review
**D3: Kyoto Encyclopedia of Genes and Genomes (KEGG)** [25]: An integrated data source consisting of several databases, broadly categorized into biological pathways, proteins, and drugs
**D4: Comparative Toxicogenomics Database (CTD)** [12]: An environmental database on chemical–protein interactions and pathway–disease relations

For the prototype, we download Bio2RDF Version 4 datasets as RDF data dumps. Each dump is deployed on an independent SPARQL endpoint locally, on a machine with 16GB RAM memory. This helps us to remove network latency and uptime of public SPARQL endpoints as issues for our experiments. The entities and relations that were extracted from each source are listed in Table 1. The SPARQL graph patterns are also presented in Table 1 to demonstrate their difference across different sources, and emphasize the need for pattern-based query federation.

We reconcile the Protein entities primarily using the HUGO Gene Nomenclature Committee (HGNC) [31] *x-ref* attributes, Drug entities using the Anatomical Therapeutic Chemical Classification [30] *x-ref* attributes, Pathway entities using KEGG *x-ref* attributes, and Phenotype entities using MESH terminology (Medical Subject Headings) *x-ref* attributes [11]. Two entities from different sources were also reconciled if *x-ref* attributes linked them to each other.

### 4.2 Inputs–Outcomes Database

During the post-marketing surveillance of drug products, the US Food and Drug Administration (FDA) collects reports on the adverse drug reactions observed in patients subjected to these drug products. The FDA Adverse Event Reporting System [15] (FAERS), a public data portal, publishes these reports after the anonymization of the patient data. As our *inputs*–*outcomes* database to learn the parameters in the model, we decided to use the FAERS datasets.

We downloaded the FAERS datasets, available as quarterly XML files, for three years from January 2013 to December 2015. Each XML file is composed of several safety reports. Among many features, each safety report indicates: *(i)* the set of adverse drug reactions observed in a patient (e.g., heart attack), and *(ii)* the set of drugs administered to the patient (e.g., Sildenafil). The string labels used by FAERS to denote the drugs and adverse drug reactions in the reports were mapped to Drug and Phenotype terms in the *k*-partite network using terminology matching methods [23] (these methods are described in more detail at http://onto-apps.stanford.edu). From an initial set of more than 3.2 million FAERS safety reports, we discarded those reports for which no Drug or Phenotype was mapped in the *k*-partite network. Hence, we were left with an aggregated dataset of around 3 million reports, with each report represented as an entry with inputs **x** = {*drug*_1_, *drug*_2_, …, *drug_m_*} and **y** = {*phen*_1_, *phen*_2_, …, *phen_k_*}.

For simplicity in probabilistic inference, each entity node in the HCRF model only has two states : −1 and 0. Depending on the type of the entity, state 1 can indicate whether a Protein or a Pathway is implicated in the association, a patient is administered a particular Drug, or he exhibits a particular Phenotype. As FAERS datasets only indicate the drugs administered and the adverse reactions observed in a patient, we do not have data on whether a particular protein or a pathway is implicated. Hence, nodes of type Protein and Pathway are hidden variables.

## 5. RESULTS

### 5.1 k-partite Network Statistics

**Figure 5:**
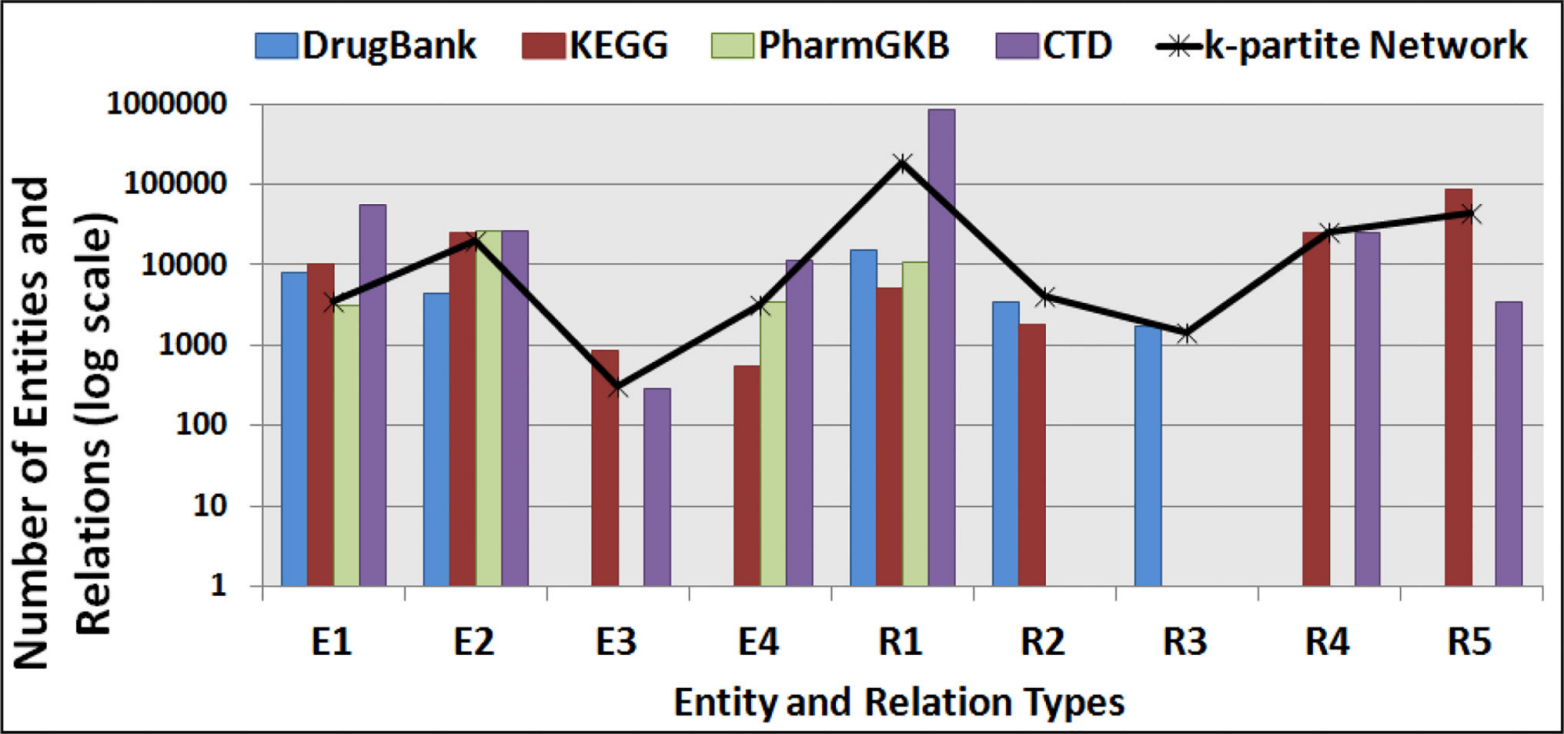
Number of Entities and Relations extracted from each data source

The number of entities and relations extracted from the four Bio2RDF Version 4 data sources by the prototype implementation of PhLeGrA are displayed in the Figure 5. The query federation module uses the SPARQL patterns (listed in Table 1). Except for CTD, PhLeGrA was able to process a given data source and retrieve the entire set of entities and relations for each type in under **2 hours**. PhLeGrA took ≈ 18 hours to process the entire CTD data source due to its size. It can be seen that the number of entities and relations for each type vary drastically across the data sources due to their granularity. Few error SPARQL patterns were discovered during this step (see **Section 6.2**).

The query federation module generated the *k*-partite network after performing entity reconciliation using the *x-ref* attributes for each entity. The largest connected component was detected in the *k*-partite network and the set of nodes that were not a part of this component were discarded. The final number of nodes in the *k*-partite network for each entity and relation type are shown in Figure 5.

**Figure 6:**
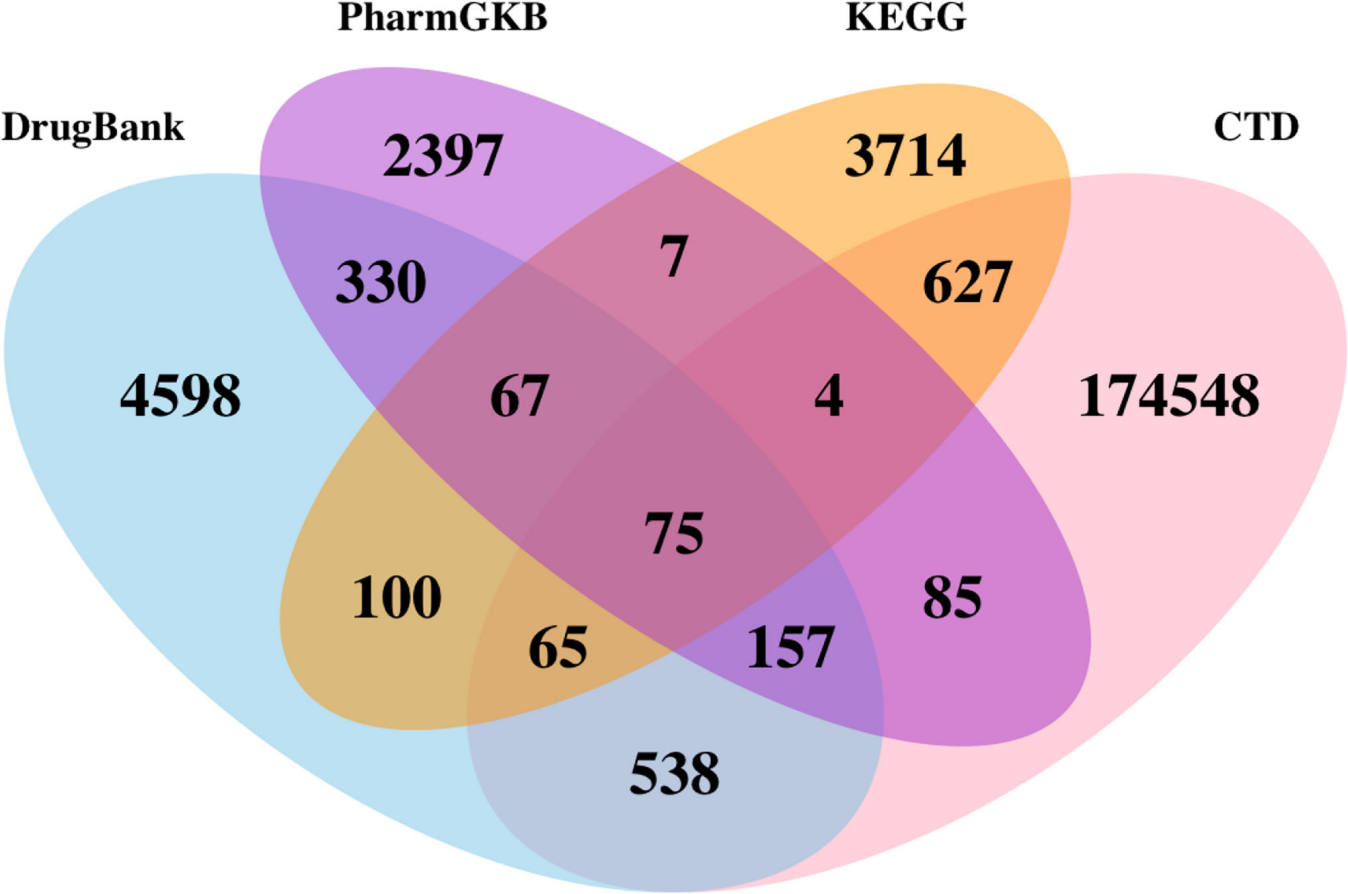
Source distribution of R1 ( Drug *hasTarget* Protein) relations. It can be seen that a majority of the R1 relations exist in only one data source.

Figure 6 depicts the source distribution of the relations of type **R1** ( Drug *hasTarget* Protein). **R1** relations are present in all four sources used in the prototype. It can be seen that a majority of these relations are unique to only one source. Hence, when generating a systems pharmacology network, query federation is beneficial if we wish to extract all possible knowledge on the drug targets. Some relations may occur in two or more sources. Hence, these relations need to be aggregated. The overlap plot also indicates that one source (CTD) may contribute, in a larger proportion, to a particular relation. This may include false-positive relations, or noise in the source, that may affect downstream association discovery. Overlap plots for other entity and relation types are available at http://onto-apps.stanford.edu/phlegra.

Using terminologies, such as ATC [30] and MESH [11], and *x-ref* attributes, is beneficial for entity reconciliation. For example, using simple entity reconciliation (reconciling entities with explicit *x-ref* links between them) the query federation module reconciled **6,043** Drug entities to **2,015** unique entities in the *k*-partite network. Using the terminologies, we were able to add an extra reconciliation step. The module further reconciled **1,568** Drug entities and **714** Phenotype entities using the ATC and MESH terminologies respectively. This helps generate a systems pharmacology network with unique entities only.

Current methods in SPARQL query federation do not govern the query reformulation process using mapping rules [35]. Assembling a systems pharmacology network using these methods, from four sources, would require an exhaustive SPARQL CONSTRUCT query [32] with several TPF expressions. Our method requires a small domain-specific data model (Figure 4) and reformulates the queries according to the mapping rules (Table 1) provided.

### 5.2 Predicting Adverse Drug Reactions

As described in **Section 3.3**, we generated an HCRF model from the *k*-partite network. We were able to identify 3,543 unique drugs and 3,186 unique ADRs in the FAERS datasets. The graph analytics module in the PhLeGrA platform can perform probabilistic inference over the entire HCRF network by taking ≈ 30 seconds for each iteration.

As a proof of concept, in this paper, we will only use 100 drugs in the HCRF network to discover new associations ({*Drug*}_*n*_ → *ADR*) efficiently. Using this small set of 100 drugs, the graph analytics module only takes ≈ 1 – 2 seconds on each iteration for training and ≈ 0.5 second for prediction. To build this set, we selected the top 20 drugs with the highest number of occurences in the FAERS dataset, and the top 80 co-mentioned drugs. Using this concise set, we were able to reduce the number of Phenotype entities to only 1276. The number of FAERS samples reduced to ≈ 0.3 million for our set of 100 drugs.

After training the HCRF using a 5-fold cross validation approach and a step size *α* of 0.01, we evaluated the trained HCRF model to predict adverse drug reactions for a combination of drugs for a separate test set. As there is no established gold standard for ({*Drug*}_*n*_ → *ADR*) associations, we created a “silver standard” test set. We held out 10,000 observations from FAERS and selected those observations with an (*Observed/Expected*) ratio greater than 2. We calculated the true and false positive rates and generated the receiver operating characteristic (ROC) curves for each Phenotype entity. We also generate a combined curve to check if we can predict each and every *outcome* using the same probabilistic threshold.

**Figure 7:**
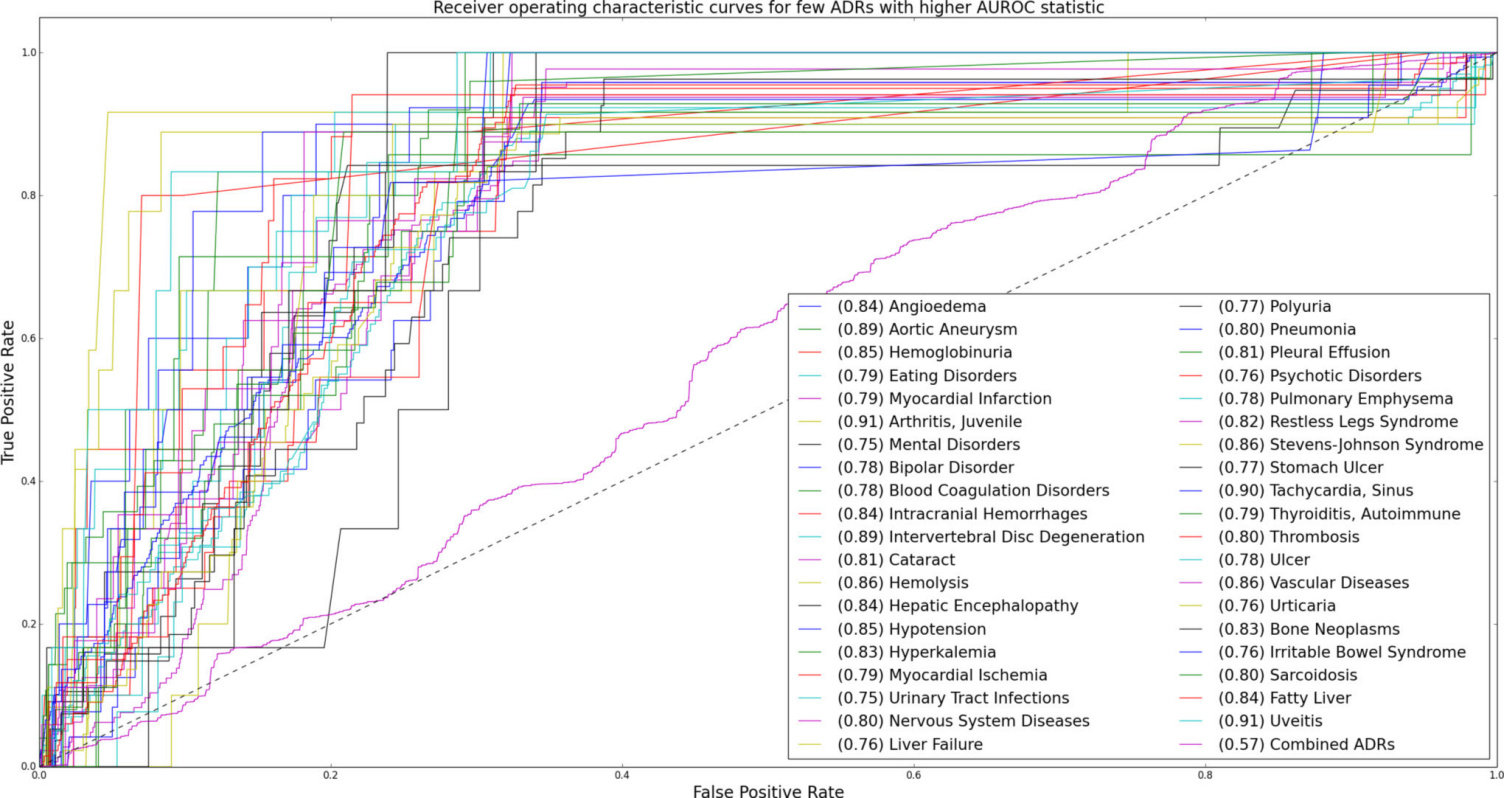
The Receiver-Operating Characteristic Curves observed during the predictions of different adverse drug reactions (ADRs) and a combination curve for the joint prediction of ADRs using the same threshold. The legends indicate the labels of the ADRs as well as the Area under the curve statistic (AUROC) for each curve (in parentheses). It can be seen that using same probabilistic threshold for every ADR results in a weaker predictive power. The HCRF model performs remarkably well to predict individual ADRs using event-specific threshold (with 146 ADRs with AUROC >= 0.75)

The area under the ROC curve (AUROC) statistic while using the same probabilistic threshold for each *outcome* is 0.57, which is barely above random guessing. However, the AUROC statistic for individual Phenotype prediction is very high for some entities, which include common adverse drug reactions such as liver failure, ulcers, polyuria, hypotension and aortic aneuryms as well as indications such as bipolar and nervous system disorders and myocardial ischemia. Figure 7 shows some of the Phenotype entities that had a higher AUROC statistic. Out of 1276 entities in the Phenotype class, 681 entities were observed in the test dataset. The HCRF model predicts 560 entities with an AUROC >= 0.5 and 146 of them with an AUROC >= 0.75.

To summarize, using the same probabilistic threshold, to predict whether an *outcome* (ADR) will result from a set of *inputs* (drugs), results in a weaker predictive power. However, the model had desirable predictive power while using event-specific thresholds for individual ADRs. The importance of event-specific thresholds for signal detection using spontaneous reporting systems such as FAERS, or electronic medical records is also highlighted previously [19]. The AUROCs obtained through our method compare favorably with these methods for the ADRs listed in Figure 7. However, our method is able to generate a probabilistic score for associations that involve more than two drugs. Moreover, exploring the probability distributions over the hidden nodes ( Protein and Pathway) may provide an insight in the underlying biological mechanisms.

### 5.3 PhLeGrA Drug–Reaction Visualizer

We developed a simple Web-based search application that allows the user to provide a set of Drugs and Phenotypes (which will be positive in our *inputs* and *outcomes* vector), and that visualizes all the possible paths that include the given drugs and the adverse outcomes. The *k*-partite network is searched iteratively by hopping across each node, and these paths are buffered back to the client, and are gradually displayed. Provenance information is displayed on hovering over the node, to list the set of data sources that the node is present in. The application can be accessed at http://onto-apps.stanford.edu/phlegra.

**Figure 8:**
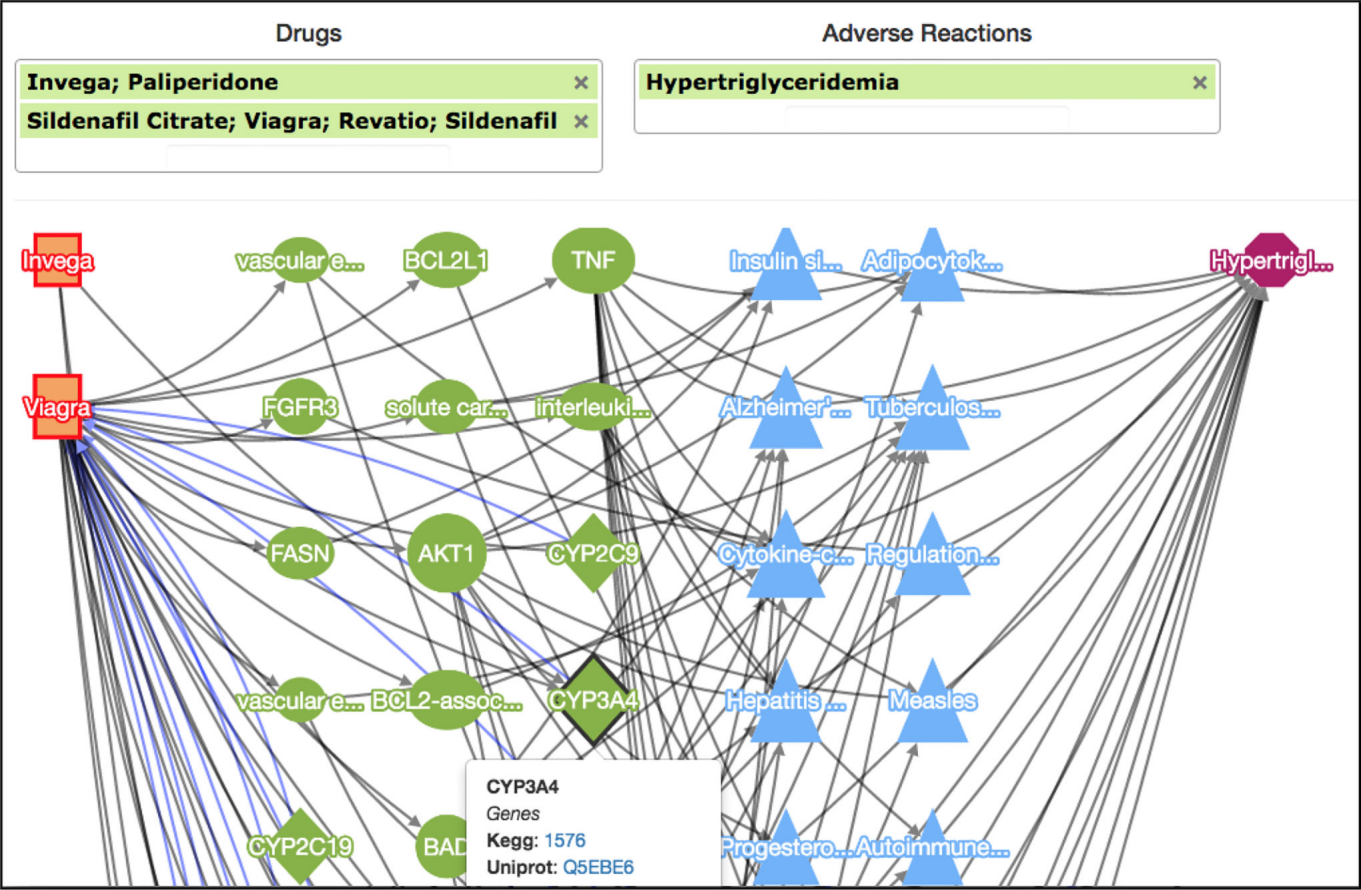
PhLeGrA Drug–Reaction Visualizer. Here, Paliperidone targets the enzymes of Sildenafil that might lead to Hypertriglyceridemia.

A screenshot of this application is shown in Figure 8. The example demonstrated in this screenshot indicates a possible DDI between Paliperidone (Invega), a drug that is used to treat bipolar disorder, and Sildenafil (Viagra), a drug that is used to treat erectile dysfunction. We observed a higher association score for ADRs such as Hypertriglyceridemia and Erectile Dysfunction that indicate reduced effects of Sildenafil. It can be hypothesized that this might be because Paliperidone inhibits Cytochrome P450 3A4 (CYP3A4) and other enzymes responsible for the metabolism of Sildenafil.

## 6. DISCUSSION

### 6.1 Linked Graph Analytics

In this paper, we present a systems pharmacology-based approach using Semantic Web technologies and query federation. We believe that generating such systems networks is extremely fast and easy using the methods presented here, as compared to traditional approaches like *CauseNet* [28] where data conversion, data integration and entity reconciliation is manual and not scalable.

We also demonstrate the benefit of query federation and entity reconciliation using a domain-specific data model and terminologies. Specifically, pattern-based query federation can be shown to bring together pharmacological knowledge existing in isolated, heterogeneous sources without being concerned about the underlying semantics and schema differences. The mapping rules still need to be assembled by the user from the SPARQL query patterns observed in the sources. An automated way to learn these query patterns and mapping rules should be explored in the future. Entity reconciliation using terminologies can enable seamless data and knowledge integration from these sources. It was observed that the LSLOD sources may sometimes not have explicit *x-ref* links between similar entities, when these entities are mapped to the same term in a terminology.

In this research, we have not incorporated more complex features of entities (e.g., molecular weight or structure of the drug) and the network should necessarily be *k*-partite (no inter-edges between nodes in the same layer). This was for simplicity to perform approximate inference on the graph. However, most real world domains, including the pharmacological domain, will not follow this straight approach. For example, two proteins may be active in only particular, disparate organs and, hence, may be independent of each other. Our current representation (Figure 4) will not be able to take into account these constraints.

However, we will argue that the PhLeGrA platform can flexibly incorporate other data and knowledge sources published using Semantic Web technologies. With modifications to the data model and the addition of newer mapping rules, different kinds of systems networks can be generated. The PhLeGrA platform be configured to use other graph analytics frameworks over these pharmacological networks. In the future, we will evaluate the utility of the PhLeGrA platform for users in the pharmacological domain.

### 6.2 Challenges using the LSLOD Cloud

Whereas linked data have been used for integrated information retrieval [22] and interactive visualization dashboards that present faceted perspectives to a knowledge base [24], they provide an opportunity to build complex machine learning models over multiple data sources. However, in the current state of the LSLOD cloud, if a user outside the Semantic Web research community wishes to utilize this integrated graph, it is very taxing. Most of the usable linked data rest as RDF data dumps in localized silos, whereas the LSLOD cloud is structurally broken (unavailable SPARQL endpoints, incorrect links and malformed URIs) and very heterogeneous (different SPARQL patterns) [37].

PhLeGrA’s query federation module relies on the set of SPARQL mappings and Endpoints for navigating the LSLOD cloud. As can be seen in Table 1, the LSLOD cloud is very heterogeneous and there is no single SPARQL graph pattern to get a simple link between a drug and its target protein. The entire potential of Semantic Web technologies rests on the idea that a naive domain user can query multiple sources regardless of the underlying heterogeneity in the schemas. However, simply extracting these links from two sources requires the end user to know the graph patterns in them. These complications increase as we retrieve additional features of an entity (e.g., molecular weight).

The quality of the LSLOD cloud sometimes necessitates several manual interventions during automated analysis. Some of the errors found empirically are listed in Table 2. These errors, while seemingly trivial, may affect query federation and information retrieval. These errors may have propagated when the representation of the identifiers in the underlying data sources changed, and automated RDF conversion pipelines were not able to capture them.

Hence, there are still several problems with the “Semantic Web” vision and the LSLOD cloud that need to be mitigated before such methods are applied to address complex, biomedical challenges like systems pharmacology.

## 7. CONCLUSION

In this research, we present the PhLeGrA platform — Linked Graph Analytics in Pharmacology. While Semantic Web technologies have been used to link heterogeneous biomedical datasets and to create the Life Sciences Linked Open Data cloud, discovering hidden associations from these linked datasets serendipitously is still an illusive goal. Through PhLeGrA, we attempt to address the the major requirements of association discovery from linked data — *i)* entity reconciliation, *ii)* query federation and *iii)* analytics. As a proof of concept, we demonstrate the utility of PhLeGrA to create a systems pharmacology network using pattern-based query federation, and to associate adverse drug reactions with drug–drug interactions using Hidden Conditional Random Field. Using event-specific thresholds, we obtained an AUROC statistic of more than 0.75 for 146 reactions. We believe that addressing the quality, availability, and heterogeneity issues in the LSLOD cloud will help improve the efficiency of the entire association discovery process and increase the utility of linked data for the domain users.

## Figures and Tables

**Table 1:** The type of entities and relations, and the SPARQL patterns, observed in each data source used in our prototype are listed below.

Source	Entity/ Relation	SPARQL Pattern
**D1**	**E1, E2**	$E1\overset{\text{drug}}{\leftarrow }\text{Target-Relation}\overset{\text{target}}{\to }E2$
	**R1, R2, R3**	$E1\overset{\text{drug}}{\leftarrow }\text{Enzyme-Relation}\overset{\text{enzyme}}{\to }E2$
		$E1\overset{\text{drug}}{\leftarrow }\text{Transporter-Relation}\overset{\text{transporter}}{\to }E2$

**D2**	**E1, E2, E4**	$E1\overset{\text{drug}}{\leftarrow }\text{gene-drug-Association}\overset{\text{gene}}{\to }E2$
	**R1**	$E2\overset{\text{gene}}{\leftarrow }\text{gene-disease-Association}\overset{\text{disease}}{\to }E4$

**D3**	**E1, E2**	$E1\overset{\text{target}}{\to }:\_\text{blank}\overset{\text{link}}{\to }E2$
	**E3, E4**	$E1\overset{\text{metabolism}}{\to }:\_\text{blank}\overset{\text{link}}{\to }E2$
	**R1, R2**	$E2\overset{\text{pathway}}{\to }E3$
	**R4, R5**	$E3\overset{\text{disease}}{\to }E4$

**D4**	**E1, E2**	$E1\overset{\text{chemical}}{\leftarrow }\text{Chemical-Gene-Association}\overset{\text{gene}}{\to }E2$
	**E3, E4**	$E2\overset{\text{pathway}}{\to }E3$
	**R1, R3, R4**	$E4\overset{\text{pathway}}{\to }E3$

**Table 2:** Error patterns found empirically

Source	Error type	Expected	Observed
**D2**	Parse Error	go:0030307	go:0030307\"
**D3**	Incorrect URIs	kegg:map00010	kegg:00010
	Capitalization	kegg:HSA_2147	kegg:hsa_2147
	Aggregated URI	kegg:HSA_1551 kegg:HSA_1576	kegg:HSA_1551 1576

## Footnotes

1 ***Phlegra*** is a spider genus of the Salticidae family, commonly termed *jumping spiders*.

2 We use the generic term ‘proteins’ to represent different biological concepts, such as ‘genes’, ‘proteins’, ‘enzymes’ and ‘transporters’ throughout this paper.
